# Supplementary material for: A simplified SARS-CoV-2 detection protocol for research laboratories
Source: PLoS One. 2020 Dec 18;15(12):e0244271. doi: 10.1371/journal.pone.0244271 (PMC7748277; doi:10.1371/journal.pone.0244271)
Supplement: Attachment — Submitted filename: Response to Reviewers-PLOS 2020.docx [file pone.0244271.s001.docx]

Response to Reviewers:

In the marked revised version of the manuscript the modifications we highlighted in yellow the text modified in response to the reviewer’s comments.

Reviewer #1

As suggested by reviewer #1 we revised minor typos throughout the manuscript

Reviewer #2

There were no specific critiques to address

To the question: Is the manuscript technically sound, and do the data support the conclusions?

The reviewer answered NO but no specific reasons were given. No specific reasons were given on the questions Has the statistical analysis been performed appropriately and rigorously? Answer No.

As for the comment “*There is nothing new in this paper because RNA extraction using the Trizol is known long before. The novelty in the paper is not impressive and it does not reveal high scientific value.*” We appreciate the comment of the reviewer although PLOSone evaluate submitted manuscripts on the basis of methodological rigor and high ethical standards, regardless of perceived novelty. Nevertheless the application of a shortened TRIzol RNA extraction protocol to nCoV2 samples from multiple clinical sources had not been described in details at the time of our submission, nor it was described the effect of TRIzol in preserving specimen at 4C or the fact that viral preparation and not viral RNA should be used for contrived samples in this type of studies. The fact that we have utilized the protocol here described to test successfully test over 1000 primary care workers (100% sensitivity and specificity to date) and I have received requests for this detailed protocol by a dozen other labs is confirming the overall quality of the work we carried out in setting up a useful nCoV2 testing protocol.

Reviewer #3

As suggested by the reviewer we updated the epidemiological data in the introduction.

We added the method utilized in the abstract.

We moved two descriptive paragraphs from the results to the introduction (from sub-heading: A simplified TRIzol protocol for the extraction of viral and cellular RNA and from sub-heading: Detection of SARS-CoV-2 in different types of clinical specimens).

We added a subheading to better organize the results (Contrieved saliva samples require the addition of the inactivated virus.).

We corrected the text and the figures as suggested by the reviewer.
